# Supplementary material for: Learning to Recognize Actions From Limited Training Examples Using a Recurrent Spiking Neural Model
Source: Front Neurosci. 2018 Mar 2;12:126. doi: 10.3389/fnins.2018.00126 (PMC5840233; doi:10.3389/fnins.2018.00126)
Supplement: Supplementary file 3 [file Presentation1.pdf]

Supplementary Material: Learning to Recognize Actions from Limited Training Examples Using a Recurrent Spiking Neural Model

Priyadarshini Panda [1,*] **and Narayan Srinivasa** [2,*]
*Correspondence:
P. Panda
Purdue Univeristy, School of Electrical & Computer Engineering, West Lafayette, IN, USA, 47906
pandap@purdue.edu

N. Srinivasa: nara@etacompute.com

DESCRIPTION: SUPPLEMENTARY VIDEO 1

The video shows the fixed threshold spike difference and the varying threshold weighted spike difference patterns obtained from a raw pixel video for BabyCrawling class using the pixel motion detection technique. It can be seen that the constant background gets eliminated and only the moving pixels (the baby in this case) are captured in the spike patterns. For each frame of the original video, a corresponding spike pattern is produced. We can also observe that the weighted spike pattern besides capturing the edges relevant to the crawling action also incorporates subtle movements such as baby's facial expression change. Towards the end of the video, we see that some background activity due to unsteady camera movement is also captured.

DESCRIPTION: SUPPLEMENTARY VIDEO 2

The video shows the various Center, Right, Left, Top, Bottom (CRLTB) scans obtained from the weighted spike pattern video for the BabyCrawling example shown in Supplementary Video 1 using the scan based filtering technique. It is clearly seen that each scan captures different relevant parts of the moving object per frame. Note, the dimensionality of each scan is 41×41 that is much smaller than the original 200×300 weighted spike image. The creation of the bounding box eliminates a large portion of the non-spiking (black) background activity thereby reducing the overall computational complexity. Furthermore, the striding of the bounding box along different directions ensures that all significant action signatures (for instance, baby's head movement/ hand gesture) are captured for a given frame across different scans. We also see that in most frames the scans only capture the baby's features while eliminating the irrelevant background activity occuring in the original spike video. However, in frames (Frame 161-164) where background activity dominates, while some portion of such clutter activity is seen in certain scans (Top, Right, Left), the remaining scans (Center, Bottom) still capture the relevant moving edges corresponding to the baby.

1: Babycrawling 65%	26: ThrowDiscus 91.6%	51: Biking 83.4%	76: CricketBowling 53.9%
2: BalanceBeam 89%	27: Typing 95.8%	52: FloorGymnastics 94.5%	77: HulaHoop 92.1%
3: BenchPress 87.5%	28: WallPushUps 94.4%	53: PoleVault 94.6%	78: Rafting 58%
4: CliffDiving 58.2%	29: HandStandWalking 80.3%	54: ShotPut 91%	79: CricketShot 74.1%
5: Diving 85.4%	30: Bowling 91.2%	55: SoccerPenalty 96.5%	80: BreastStroke 78.9%
6: GolfSwing 97%	31: Punch 56%	56: BlowDryHair 61%	81: Drumming 79.2%
7: HammerThrow 94.2%	32: Billiards 89%	57: Surfing 94.3%	82: HeadMassage 55.8%
8: HandStandPushups 89.8 %	33: HorseRace 56.1%	58: Fencing 70.1%	83: BrushingTeeth 71.1%
9: LongJump 94.2%	34: TrampolineJumping 62%	59: Skiing 79.8%	84: BaseballPitch 79.3%
10: BodyWeightSquats 58.3%	35: PlayingTabla 78%	60: Rowing 78.4%	85: BlowingCandles 77%
11: JumpingJack 94.8%	36: BasketBallDunk 63%	61: TableTennisShot 77.3%	86: TaiChi 94.7%
12: JumpRope 65%	37: IceDancing 77%	62: FrontCrawl 94.3%	87: WalkingWithDog 73.7%
13: CleanandJerk 94.5%	38: Skijet 86.3%	63: SumoWrestling 67.3%	88: ApplyLipstick 78.3%
14: PlayingCello 88%	39: VolleyballSpiking 62%	64: YoYo 64.1%	89: CuttingInKitchen 92.9%
15: PlayingGuitar 63.4%	40: SkyDiving 66.4%	65: BandMarching 79.4%	90: RopeClimbing 93.4%
16: PlayingPiano 95%	41: BoxingPunchingBag 88%	66: FrisbeeCatch 68.3%	91: ShavingBeard 72.5%
17: PlayingFlute 79.6%	42: HorseRiding 93.7%	67: RockClimbingIndoor 78.4%	92: PizzaTossing 89.4%
18: PlayingDhol 61.8%	43: SalsaSpin 48%	68: Mixing 79.5%	93: Hammering 84%
19: ParallelBars 89%	44: WritingonBoard 79.2%	69: JugglingBalls 81.4%	94: Archery 91.3%
20: PlayingViolin 91.1%	45: PlayingSitar 92.3%	70: MilitaryParade 58.3%	95: BasketBall 96.2%
21: PommelHorse 97%	46: Knitting 61.1%	71: Kayaking 91.3%	96: Nunchuks 88.6%
22: Pullups 80%	47: BoxingSpeedBag 82%	72: FieldHockeyPenalty 93%	97: MoppingFloor 86.1%
23: Pushups 78%	48: Swing 79%	73: PlayingDaf 95%	98: Lunges 94%
24:UnevenBars 89.6%	49: StillRings 80.8%	74: ApplyEyeMakeup 77.2%	99: JavelinThrow 93.2%
25:TennisSwing 59.4%	50: SkateBoarding 62.5%	75: SoccerJuggling 57.2%	100: HighJump 93%
			101: HairCut 73.4%

Figure S1. Class-wise accuracy obtained with 8000 neuron (10% connectivity) model on UCF-101 dataset when trained with 8 training videos per class. Few select classes with high/low accuracy are highlighted with green/red font to quantify the classes with more/less consistent videos.

1 SUPPLEMENTARY DATA

D/A model for Action Recognition

Fig. S1 shows the class-wise accuracy obtained with our reservoir approach on UCF101 dataset. We can deduce that certain classes are learnt more accurately than the others. This is generally seen for those classes that have more consistent action signatures and lesser variation such as *GolfSwing, PommelHorse* among others. On the other hand, classes that have more variations in either action or views or both, such as *TennisSwing, CliffDiving* etc yield less accuracy. In fact, classes that consist of many moving subjects such as *MilitaryParade, HorseRacing* exhibit lower accuracy. In such cases, the input processing technique may not be able to extract motions from all relevant moving subjects. Here, irrelevant clutter or jitter due to camera motion gets captured in the spiking information as a result of which the reservoir is unable to recognize a particular signature. Such limitations can be eliminated with an adequate processing technique that incorporates depth and (or) motion based tracking. Also, data augmentation with simple transformations can further improve the accuracy. For instance, for a given training video, incorporating both left/right handed views by applying a $180\deg$ rotational transformation to all the frames of the original video will yield a different view/action for the same video. Such data augmentation will account for more variations in views/angles enabling the reservoir to learn better.

Fig. S2 shows the confusion matrix of the 101-class problem for the same reservoir topology as Fig. S1.

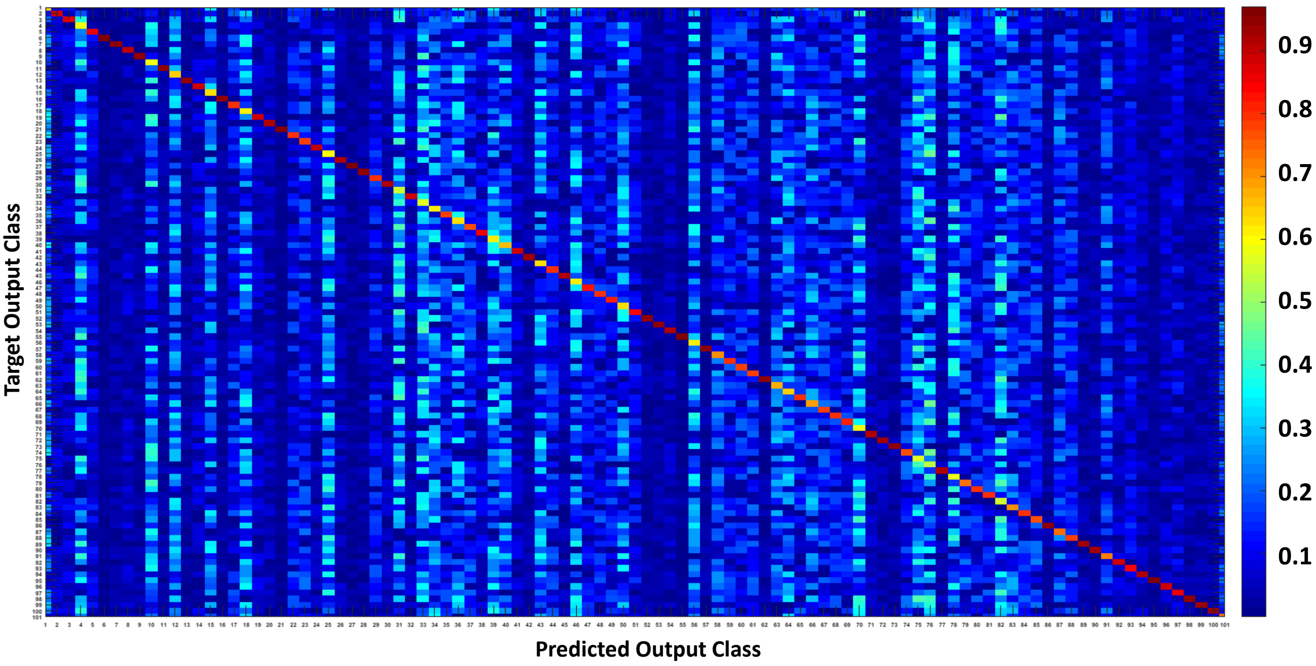

Figure S2. Confusion Matrix for 101-class problem corresponding to Fig. S1 above. The X/Y axis represent the class numbers with equivalent notations as that of Fig. S1. The colormap indicates the accuracy for a given class label with highest intensity (red) corresponding to 100% match between target & output class and lowest intensity (blue) corresponding to 0% match between target & output class.

(a)

VGG-16 (# Tunable Parameters 97.5M)

# Training Examples	Top-1 (%)	Top-3 (%)	Top-5 (%)
Full Training Data	66.2	81.6	89.8
8 videos per class	42.9	49.7	53.4
40 videos per class	50.02	56.3	65.2

(b)

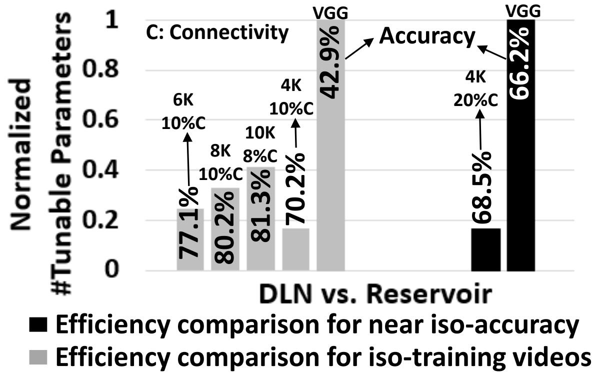

Figure S3. (a) Top-1/3/5 accuracy obtained with the VGG-16 deep learning model on 101-classes for different training scenarios (b) Efficiency (quantified as total # of tunable parameters) comparison between VGG-16 model and D/A reservoir model of different topologies (from Fig. 8 (a) in main manuscript) for iso-accuracy and iso-data (same number of training examples) scenario. The accuracy/topology for each model is noted on the graph.

Here, we further quantify the advantages with our reservoir approach against the spatial VGG-16 model described in the main manuscript by gauging the efficiency vs. accuracy tradeoff obtained from both the models in equivalent training scenarios. Fig. S3 (a) illustrates the accuracy obtained for different training scenarios. For scenario 1, wherein the entire training dataset is used, the Top-1 accuracy obtained is 66.2%, thus implying the detrimental effect of disregarding temporal statistics in the data. Then, we tested the VGG model for two different limited example scenarios, one equivalent to our reservoir learning with 8 training examples per class and other with 40 training examples. In both cases, we see that the accuracy

drops drastically with the 8-training example scenario yielding merely 42.9% accuracy. Increasing the number of training examples to 40 does improve the accuracy to 50.02%, but the performance is much lower than that of the reservoir model.

A noteworthy observation in Fig. S3 (a) is that the Top-5 accuracy for the VGG-16 model (89.8%) trained with the entire dataset and the 8000N-10% connectivity reservoir (86.1%) trained on 8 videos per class (refer to Fig. 8 (a) in main manuscript) is comparable. In contrast, for limited training scenarios, the Top-5 accuracy with VGG is drastically reduced as compared to the reservoir models shown in Fig. 8 (a) of the main manuscript. This is indicative of the DLN's inferior generalization capability on temporal data.

Fig. S3 (b) shows the normalized benefits observed with the different topologies of reservoir (Fig. 8 (a) in main manuscript) as compared to spatial VGG-16. Here, we quantify efficiency in terms of total number of trainable parameters (or weights) in a given model. The VGG-16 model for 101-class has 97.5M tunable parameters, against which all other models in Fig. S3 (b) are normalized. We observe $\sim 6\times$ improvement in efficiency with the reservoir model where both VGG and the reservoir (*4000-N reservoir with 20% connectivity*) have almost similar accuracy $\sim 67\%$. A noteworthy observation here is that VGG-16 in this case was trained with the entire UCF101 training data, while our reservoir had only 8 samples per class for training. This ascertains the potential of reservoir computing for temporal data processing. In an iso-data scenario (i.e. 8 videos per class for both VGG and reservoir models), we observe that the reservoirs yielding maximal accuracy of $81.3\%(80.2\%)$ continue to be more efficient by $2.2\times(3\times)$ than the VGG model.

For implementing the VGG-16 convolutional network model, we used the machine learning tool, Torch Collobert et al. (2011). Also, we used standard regularization Dropout Srivastava et al. (2014) technique to optimize the training process for yielding maximum performance. In case of the DLN model, we feed the raw pixel valued RGB data as inputs to the DLN for frame-by-frame training thereby providing the model with all information about the data. The input video frames originally of 200×300 dimensions are resized to 224×224. The macro-architecture of the VGG-16 model was imported from Zagoruyko (2015) and then altered for 101-class classification. To ensure sufficient number of training examples for the deep VGG-16 model in the limited example learning cases, we augmented the data via two random transformation schemes: rotation in the range of $(0 - 40\,\mathrm{deg})$, width/ height shifts within 20% of total width/height. For the 8/40-training example scenario, each frame of a training video was augmented 10/2 times, respectively using any of the above transformations. The VGG-16 model in each of the training example scenarios described above is trained for 250 epochs. Here, in each epoch, the entire limited/full training dataset (that is the individual frames corresponding to all classes) are presented to the VGG model. Note, UCF101 provides three train/test split recommendations. We use just split #1 for all of our experiments.

D/A model for Speech Recognition

Till now, we have discussed the application of our D/A based reservoir model on action recognition. However, it is evident that the methods presented here go beyond images/videos. To illustrate the effectiveness of our methodology on a different domain, we verified the model on a speech classification task for spoken digits on a subset of the NIST TI46 speech corpus dataset. The subset contains a total of 500 speech samples that includes 10 utterances each of digits 0-9 spoken by 5 different speakers, and is de facto used in existing works to evaluate models for speech recognition Torrejon et al. (2017); Verstraeten et al. (2005). The speech recordings were pre-processed based on the Lyons Passive Ear model Lyon (1982) of the human cochlea using Slaneys MATLAB auditory toolbox Slaney (1998). The Lyons cochlear model extracts the time evolution of frequency channels characterizing a speech signal. At any given time, the

intensity of different frequency channels are mapped to the instantaneous firing rate of the corresponding input neurons. We use a Poisson process to generate input spikes based on the individual neuronal firing rates constrained between 0 and 64Hz. In our experiment, each speech sample is uniquely represented by the time evolution of 39 frequency channels that constitute the input neurons.

Fig. S4 illustrates the accuracy obtained for different topologies of the reservoir model with varying number of training examples. Here, unlike the action recognition experiment, we only derive one Auto model from a Driven model. The Auto model is then presented with the Poisson spikes corresponding to the 39 channels obtained from the cochlear processed speech patterns. For limited training scenarios, first, we trained our model with 5 speech patterns per class uttered by 5 different speakers. Our analysis with 5 training examples per class (total $5 \times 10 = 50$ training examples) on the D/A reservoir model (1000Neurons-10% connectivity) yields 87.6% accuracy that signifies the capability of our model to classify speech instances with limited examples. The accuracy increases to 92.8% by increasing the number of training patterns to 10 per class (that includes 2 utterances per speaker per class), which is comparable to the accuracy of $\sim$93% provided by a vanilla Liquid State Machine (LSM) of 1200 neurons (with similar pre-processing front-end) Verstraeten et al. (2005) trained on the full training data. It is evident that our model owing to smaller reservoir size and significantly less training patterns than the LSM model in Verstraeten et al. (2005) is computationally more efficient. This further establishes the universality of our approach in a limted training scenario.

Note, with regard to training for the speech classification task, the Auto model is trained for 15 epochs. In each training epoch, we present the training input patterns corresponding to all classes sequentially (for instance, Class1$\rightarrow$Class 2$\rightarrow$Class 3) to produce target patterns similar to Fig. 2 (b) of the main manuscript. Each input pattern is presented for a time period of 700 ms (or 700 time steps). Also, before presenting a new input pattern, the membrane potential of all neurons in the reservoir are reset to their resting values. The testing is done on the remaining patterns (not used for training) in the spoken digit database. During testing, we present the test patterns for 5 epochs (wherein the entire test data corresponding to all classes is presented in each epoch). The prediction accuracy across 5 epochs is then averaged and then reported as the final accuracy in Fig. S4. The standard deviation of classification accuracy, in this case, across the 5 test epochs for all experiments ranges between 0.6 -1.2%.

Reservoir Model (10% Connectivity)	# Training Speech Patterns	Accuracy (%)
400	5 per class	81.2
	10 per class	86.3
600	5 per class	85.5
	10 per class	89
800	5 per class	87
	10 per class	92.1
1000	5 per class	87.6
	10 per class	92.8

Figure S4. Accuracy obtained with 5-/10-training examples (per class) for D/A models of different topologies on the TI46 speech classification task.

REFERENCES

Collobert, R., Kavukcuoglu, K., and Farabet, C. (2011). Torch7: A matlab-like environment for machine learning. In *BigLearn, NIPS Workshop*. EPFL-CONF-192376

Lyon, R. (1982). A computational model of filtering, detection, and compression in the cochlea. In *Acoustics, Speech, and Signal Processing, IEEE International Conference on ICASSP'82.* (IEEE), vol. 7, 1282–1285

Slaney, M. (1998). Auditory toolbox. *Interval Research Corporation, Tech. Rep* 10, 1998

Srivastava, N., Hinton, G. E., Krizhevsky, A., Sutskever, I., and Salakhutdinov, R. (2014). Dropout: a simple way to prevent neural networks from overfitting. *Journal of machine learning research* 15, 1929–1958

Torrejon, J., Riou, M., Araujo, F. A., Tsunegi, S., Khalsa, G., Querlioz, D., et al. (2017). Neuromorphic computing with nanoscale spintronic oscillators. *Nature 547, 10.1038/nature23011* , 428–431

Verstraeten, D., Schrauwen, B., and Stroobandt, D. (2005). Isolated word recognition using a liquid state machine. In *ESANN*. vol. 5, 435–440

Zagoruyko, S. (2015). http://torch.ch/blog/2015/07/30/cifar.html
